# Radiomics-Based OCT Analysis of Choroid Reveals Biomarkers of Central Serous Chorioretinopathy

**DOI:** 10.1167/tvst.14.4.23

**Published:** 2025-04-23

**Authors:** Ryan Chace Williamson, Kiran Kumar Vupparaboina, Sandeep Chandra Bollepalli, Mohammed Nasar Ibrahim, Nicola Valsecchi, Arman Zarnegar, José-Alain Sahel, Jay Chhablani

**Affiliations:** 1 Ophthalmology, UPMC, Pittsburgh, PA, USA; 2 University of Pittsburgh School of Medicine, Pittsburgh, PA, USA; 3 Ophthalmology, University of Pittsburgh, Pittsburgh, PA, USA

**Keywords:** radiomics, biomarkers, OCT, central serous chorioretinopathy, choroid

## Abstract

**Purpose:**

Biomarkers from choroidal imaging can enhance clinical decision-making for chorioretinal disease; however, identification of biomarkers is labor-intensive and limited by human intuition. Here we apply radiomics feature extraction to choroid imaging from swept-source optical coherence tomography (SS-OCT) to automatically identify biomarkers that distinguish healthy, central serous chorioretinopathy (CSCR), and unaffected fellow eyes.

**Methods:**

Radiomics features were extracted from SS-OCT images from healthy (n = 30), CSCR (n = 39), and unaffected fellow eyes (n = 20), with a total of 44,500 single-cross sectional horizontal images and 8900 en face images. Logistic regression classification of eyes as healthy versus CSCR, healthy versus fellow, or CSCR versus fellow was performed using radiomics features. Statistical significance was determined using 95% bootstrap confidence intervals.

**Results:**

Significant differences between healthy and CSCR eyes were found for all radiomics feature groups. Classification of health versus CSCR achieved classification accuracy of 84.2% (77.2%–89.9%) in horizontal images and 85.3% (78.2%–90.7%) in en face images. For en face images, classification accuracy increased by 1.02% (0.50%–1.53%) for every 10% increase in choroid depth. Fellow eye classification using a classifier trained to distinguish healthy and CSCR eyes resulted in 90.4% (90.2%–90.6%) of horizontal images and 90.2% (89.8%–90.2%) of en face images being classified as CSCR.

**Conclusions:**

These results demonstrate accurate classification of healthy and CSCR eyes using choroid OCT radiomics features. Furthermore, radiomics features revealed signatures of CSCR in unaffected fellow eyes.

**Translational Relevance:**

These findings demonstrate the potential for radiomics features in clinical decision support for CSCR.

## Introduction

Central serous chorioretinopathy (CSCR) is a chorioretinal disease that causes serous detachment of the macular neurosensory retina.^1^^–^^4^ Previous work using optical coherence tomography (OCT) has identified key features of the choroid in patients with CSCR including choroidal thickness^5^^,^^6^ and vascularity index^7^^,^^8^ that distinguish it from other pathologies and allow tracking of the disease over time. Despite these successes, identification of additional useful choroidal biomarkers remains a labor-intensive process. This process can be sped up using automated biomarker extraction with radiomics texture feature analysis.^9^ Although this technique has been used to identify biomarkers for retinal pathology and to study healthy choroid,^10^ to date, no study has applied radiomics techniques to choroid OCT in pathologic eyes. We address this gap by using radiomics texture analysis to extract biomarkers for the identification of eyes with CSCR.

Machine learning has become a powerful tool for feature extraction in ophthalmic diseases including age-related macular degeneration,^11^^,^^12^ diabetic retinopathy,^13^^–^^15^ and others.^16^ Limited work using choroid OCT has demonstrated the application of machine learning tools to assess image quality,^17^ predict disease course,^5^^,^^18^^,^^19^ and automatically segment anatomy.^20^ These studies demonstrate the potential of choroid OCT as a source of biomarkers for clinical decision support in a variety of contexts. Additional work is needed to understand how to best extract biomarkers that characterize the relationship between choroid architecture and chorioretinal disease.

Radiomics-based texture analysis utilizes standard image analysis techniques to extract texture features from images. These techniques have had wide application in the fields of radiology and pathology and in recent years have seen increasing application in ophthalmology to a variety of imaging modalities including OCT,^12^^,^^21^^,^^22^ OCT angiography,^10^^,^^23^ and ultra-wide field fluorescein angiography^24^^,^^25^ and to a variety of pathologies including diabetic macular edema,^24^^,^^25^ myopic maculopathy,^26^ and age-related macular degeneration.^12^^,^^21^ Limited work has studied radiomics features in healthy choroid, describing low variability of texture features across healthy individuals.^10^ Previously, our team demonstrated the use of radiomics features from pigment epithelial detachment for predicting anti-vascular endothelial growth factor treatment response.^12^ Further work is needed to assess how radiomics features differ between healthy and pathologic states in choroid.

Radiomics has multiple advantages over traditional deep learning methods when it comes to feature extraction. First, radiomics does not require a learning step and can therefore be performed with a relatively small patient sample and with limited computing power. Additionally, deep learning feature extraction parameters are learned and therefore vary between model instances even when the training data is the same. Because of this, it can be difficult to “open the black box,” decreasing the interpretability of these models.^27^^–^^29^ Because radiomics uses standardized methods, the extracted features can be studied in-depth, allowing for the development of greater interpretability.^9^^,^^16^ Radiomics is also computationally light due to the pre-defined nature of the feature transformations. At the same time, since the feature transforms can be nonlinear, it offers the same flexibility of other more computationally expensive nonlinear machine learning models. Finally, radiomics features can be input into other machine learning classification techniques, creating an interpretable and sample efficient machine learning model that can be applied in a wide range of situations.^12^

In this study, we applied radiomics-based texture analysis to extract features from OCT of choroid in healthy and CSCR patients. We first identified features that distinguished between healthy and CSCR eyes. We next applied linear classification to extracted features to study the feasibility of CSCR classification with radiomics. Next, we focused the feature and classification analyses on local tissue regions to identify which areas of choroid most distinguish between healthy and CSCR eyes. Finally, we compared CSCR and unaffected fellow eyes to identify features that may predict risk of fluid accumulation.

## Methods

### Data Acquisition

We conducted a retrospective cross-sectional study involving patients who underwent swept source OCT (SS-OCT) imaging as part of their clinical care. Informed consent was obtained for all patients. The study was conducted in accordance with the Declaration of Helsinki and the Institutional Review Board of the University of Pittsburgh.

In total 30 eyes from 25 healthy patient, 39 eyes in 32 patients with CSCR, and 20 unaffected fellow eyes from 20 of the patients with CSCR were included in this study. All patients underwent a complete history and comprehensive ophthalmic examination including visual acuity, intraocular pressure, slit lamp biomicroscopy, and dilated fundus examination to confirm the lack of pathology in healthy patients and the presence of CSCR in CSCR patients. We included patients over 18 years old with confirmed diagnosis of chronic CSCR (onset greater than three months before imaging) and with availability of comprehensive patient clinical histories and high-definition OCT images. Patients with other ophthalmic disorders, such as glaucoma or other optic nerve alterations, advanced cataracts, prior intraocular surgeries (except uncomplicated cataract surgery), vitreoretinal pathologies, uveitis, diabetic retinopathy, or other vascular disorders in either eye, high myopia (beyond −6 diopters), and any disorder that affected the retinal examination or OCT image clarity were excluded from the study. SS-OCT volumetric b-scan images were obtained for each eye included in the study using a Plex-elite 9000 wide-field SS-OCT device. Segmentation of choroid was performed using a previously described automatic segmentation algorithm.^30^ For each eye, 500 images were obtained for a total of 44500 horizontal scan images. En face images were reconstructed from b-scan volumes with generation of 100 en face slices per eye for a total of 8900 en face images.

### Feature Extractions

**Figure 1.**
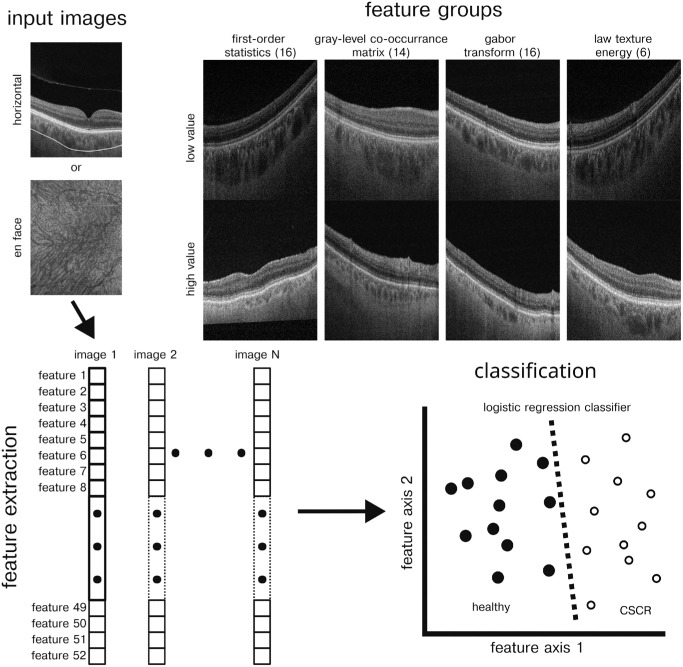
Radiomics pipeline. *Upper left*: Single cross-sectional horizontal scans are segmented to identify the region of interest. *Lower left*: Radiomics features are then extracted from the segmented horizontal scans or from en face images. *Lower right*: Extracted radiomics features are used as input to machine learning classification. *Upper left*: The radiomics features studied are separated into four groups. Example images show large and small values from example features within each of the feature groups.

Radiomics texture feature extraction was performed on b-scan and en face segmented choroid using the Pyfeats python-based radiomics library.^31^ Four categories of features were extracted: first-order statistics (FOS) features, Gray-Level Co-Occurrence Matrix (GLCM) features,^32^ Gabor texture (GT) features,^33^ and Law texture energy (LTE) features^34^ (Fig. 1). FOS features included 10th, 25th, 75th, and 90th percentile pixel values, coefficient of variation, energy, entropy, histogram width, kurtosis, maximal gray level, mean, median, minimal gray level, mode, skewness, and variance. GLCM features were summarized with the following statistics of the GLCM: angular second moment, contrast, correlation, sum of squares variance, inverse difference moment, sum average, sum variance, sum entropy, entropy, difference variance, difference entropy, information measure of correlation features, and maximal correlation coefficient. To obtain GT features, images pixels were first offset so that pixel values were between −0.5 and 0.5. Next Gabor filters with angles of 0°, 45°, 90°, and 135° and spatial frequencies of 0.1 and 0.4 cycles/pixel. GT features included Gabor filter output mean and standard deviation for each angle and spatial frequency combination. LTE features were extracted using a mask size of 3. In total, 52 features were extracted from each b-scan or en face image.

To compare features between the two groups, features were first z-scored across all images. Next, features with the greatest difference between healthy and CSCR eyes were identified using a non-paired *t*-test. Features were then ranked within each feature group. The top two features for each group were then plotted. The same procedure was performed for comparing healthy and fellow features and CSCR and fellow features. Significance for feature comparisons was determined by *p*-value from the non-paired *t*-test with Bonferroni correction.

### Image Classification

Image classification was performed in a leave-one-eye-out cross-validated manner as follows. All images from a single eye were held out as a test data set and images from all remaining eyes were used for classifier model training. Next training image features were z-scored using feature mean and standard deviation calculated from training images. Classification was then performed using a logistic regression model implemented using the sklearn python library to classify choroid features as either “healthy” or “CSCR.”^35^ Test images from the held out eye were then z-scored using the mean and standard deviation obtained from the training images. Model performance was measured using accuracy and receiver operator characteristic area under the curve (ROC-AUC). This processes was repeated separately using images from each eye as a test data set. Bootstrap 95% confidence intervals were then computed across model performance metrics obtained from each eye using 1000 bootstrap iterations. To study variation in classification with feature group, we repeated the above procedure with the exception of only including features within a given feature group for training and classification. These classifiers were also applied to fellow eye images to assess if fellow eye features in aggregate were more similar to healthy or CSCR features. Classification of healthy versus fellow and CSCR versus fellow was also performed using the procedure described above.

To study variation in choroid features across space, the above procedure was repeated for en face slices within localized regions. In other words, for each cross-validation fold, rather than using all images for a given eye, only en face slices from a section of choroid were included in the training and test sets. Specifically, images from local regions of 10% of choroid thickness were studied. Linear regression analysis was then performed on the mean classification performances for each depth to assess the trend in classification performance across layers of choroid.

## Results

A total of 30 eyes from 25 healthy patient and 39 eyes in 32 patients with CSCR were included in this study. In addition 20 unaffected fellow eyes from 20 of the patients with CSCR were also included for comparison with healthy and CSCR eyes. Average age was 50.9 (42.5–59.3, 95% bootstrap confidence interval) years for healthy patients, 51.0 (47.7–54.6, 95% bootstrap confidence interval) years for CSCR patients, and 52.2 (47.1–57.5, 95% bootstrap confidence interval) years for fellow eye patients. PLEX Elite (Zeiss, Oberkochen, Germany) swept-source OCT images were obtained from each eye, and radiomics-based texture analysis on automatically segmented choroid was performed on a total of 44,500 horizontal scan images and 8900 en face images (Fig. 1).

**Figure 2.**
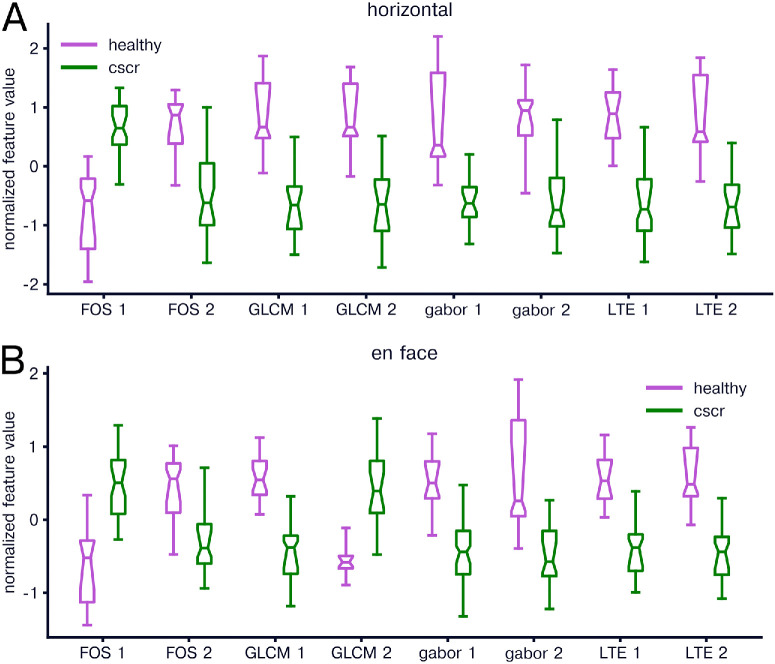
Comparison of radiomics features in healthy and CSCR eyes. (**A**) Normalized radiomics features for healthy (*magenta*) and CSCR (*green*) horizontal b-scan images. For each box-whisker plot, the central line indicates the median, the notch area indicates a bootstrap 95% confidence interval around the median, the ends of the box indicate the interquartile range, and the whiskers indicate 1.5 times the interquartile range. The two features for each feature group with the lowest *P* value when comparing healthy and CSCR are included in the plot, labeled as feature group name followed by 1 or 2. (**B**) Same as in A but for en face images.

We first compared texture features between healthy and CSCR eyes. We found significant differences between healthy and fellow horizontal images in 13 of 16 FOS features, 12 of 14 GLCM features, 15 of 16 Gabor features, and six of six LTE features. The top two texture features in each feature group for horizontal scans were for FOS features FOS skewness and FOS mode, for GLCM features contrast mean and difference entropy, for Gabor features angle 135/spatial frequency 0.1 standard deviation and angle 90/spatial frequency 0.4 mean, and for LTE features LE and LS (Fig. 2A). For en face images, we found significant results for 13 of 16 FOS features, 11 of 14 GLCM features, 12 of 16 Gabor features, and five of six LTE features. The top two texture features in each feature group for en face scans were for FOS features FOS skewness, and FOS mode, for GLCM features difference entropy mean and inverse difference moment mean, for Gabor features angle 45/spatial frequency 0.1 mean and angle 135/spatial frequency 0.4 standard deviation, and for LTE features EE and ES (Fig. 2B).

**Figure 3.**
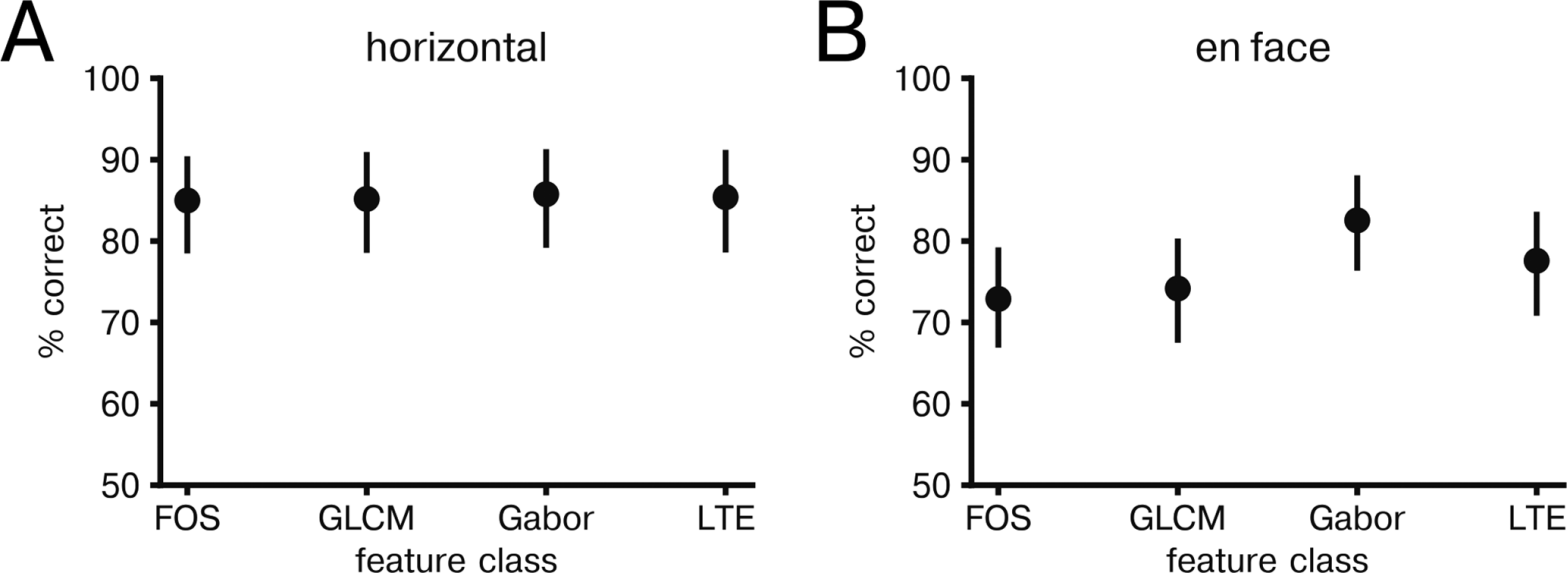
Classification of healthy versus CSCR by feature group. (**A**) Mean classification accuracy by feature group for horizontal images. Error bars indicate bootstrap 95% confidence interval over held out eyes (N = 69). (**B**) Same as in A, but for en face images.

To determine whether feature differences could be used for classification of healthy and CSCR eyes based on OCT of choroid, we applied a logistic regression model to texture features. When applied to horizontal scans, logistic regression classification produced a classification accuracy of 84.2% (77.2%–89.9%) and ROC-AUC 0.923 (0.846–0.979). Logistic regression applied to en face images resulted in a classification accuracy of 85.3% (78.2%–90.7%) and ROC-AUC 0.951 (0.891–0.989). To rule out spurious results, a shuffle control was used in which image class label was shuffled across eyes with resulting accuracy of 52.9% (45.6%–60.6%) and ROC-AUC of 0.532 (0.370–0.677) for horizontal scans and 50.8% (43.7%–58.9%) and ROC-AUC of 0.518 (0.372–0.661) for en face scans. We then evaluated how classification accuracy depended on feature group. In horizontal scans, we found no significant difference in prediction accuracy across feature groups (Fig. 3A). However, in en face images we found that Gabor features had significantly higher classification accuracy compared to all FOS and GLCM features (*P* value < 0.05, paired two-tailed *t*-test; Fig. 3B). No other feature groups showed significant differences in classification accuracy of en face images.

**Figure 4.**
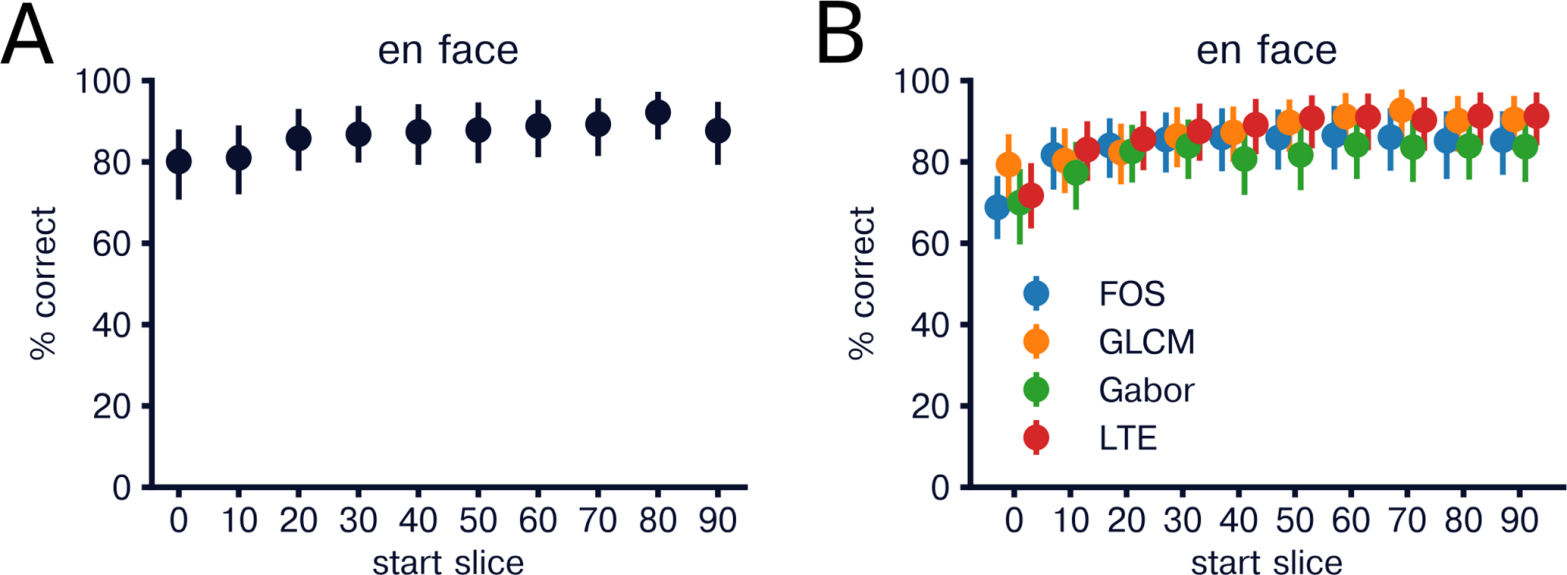
Classification of healthy vs CSCR by en face depth in choroid. (**A**) Mean classification accuracy of healthy versus CSCR using all features separated by slice depth. For each classification, 10 consecutive en face images were used from each eye starting at the “start slice.” *Error bars* indicate bootstrap 95% confidence interval over held out eyes (N = 69). (**B**) Same as in A but classification was performed using only features from a single feature group. Performances for each group at each depth are indicated.

Next, we evaluated how features varied with depth within the choroidal vasculature. To do this we evaluated texture features in en face images of healthy and CSCR patients and compared classification accuracy in 10% increments. We found that average classification accuracy increased with depth with linear regression analysis demonstrating an average increase in classification accuracy of 1.02% (0.50%–1.53%) for every ten percent increase in depth (Fig. 4A). We then evaluated whether classification accuracy variation across depth depended on feature group. We found significant increases in accuracy for Gabor, GLCM, and LTE features with classification accuracy increase per 10% depth of 1.04% (0.24%–1.84%), 1.38% (0.88%–1.88%), and 1.6% (0.71%–2.57%), respectively (Fig. 4B). FOS features slope did not reach significance, but demonstrated a trend with increasing accuracy per 10% depth of 1.11% (−0.00% to 2.22%).

**Figure 5.**
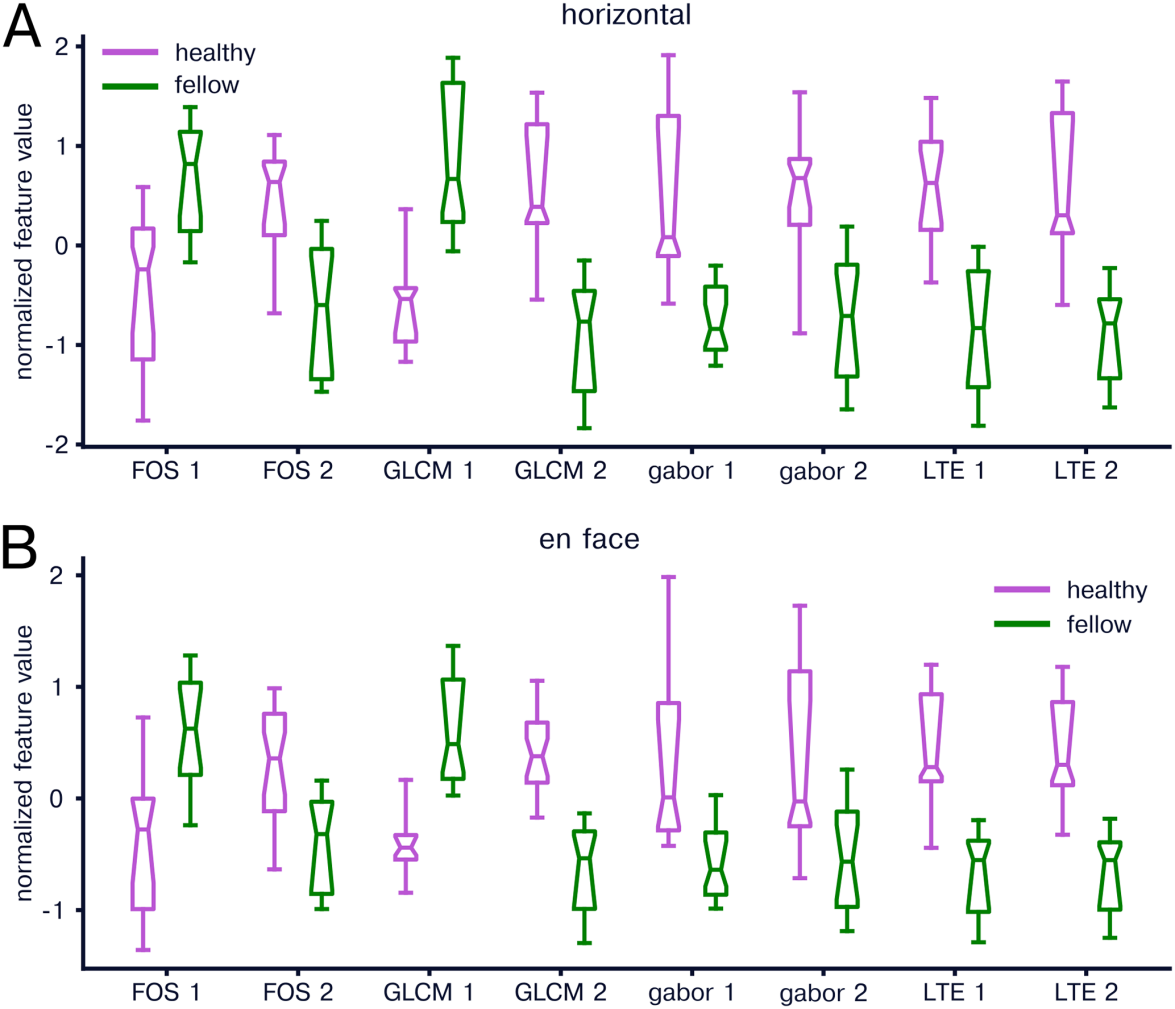
Comparison of radiomics features in healthy and fellow eyes. (**A**) Same as in Figure 1A but comparing healthy and fellow eyes. (**B**) Same as in Figure 1B but comparing healthy and fellow eyes.

**Figure 6.**
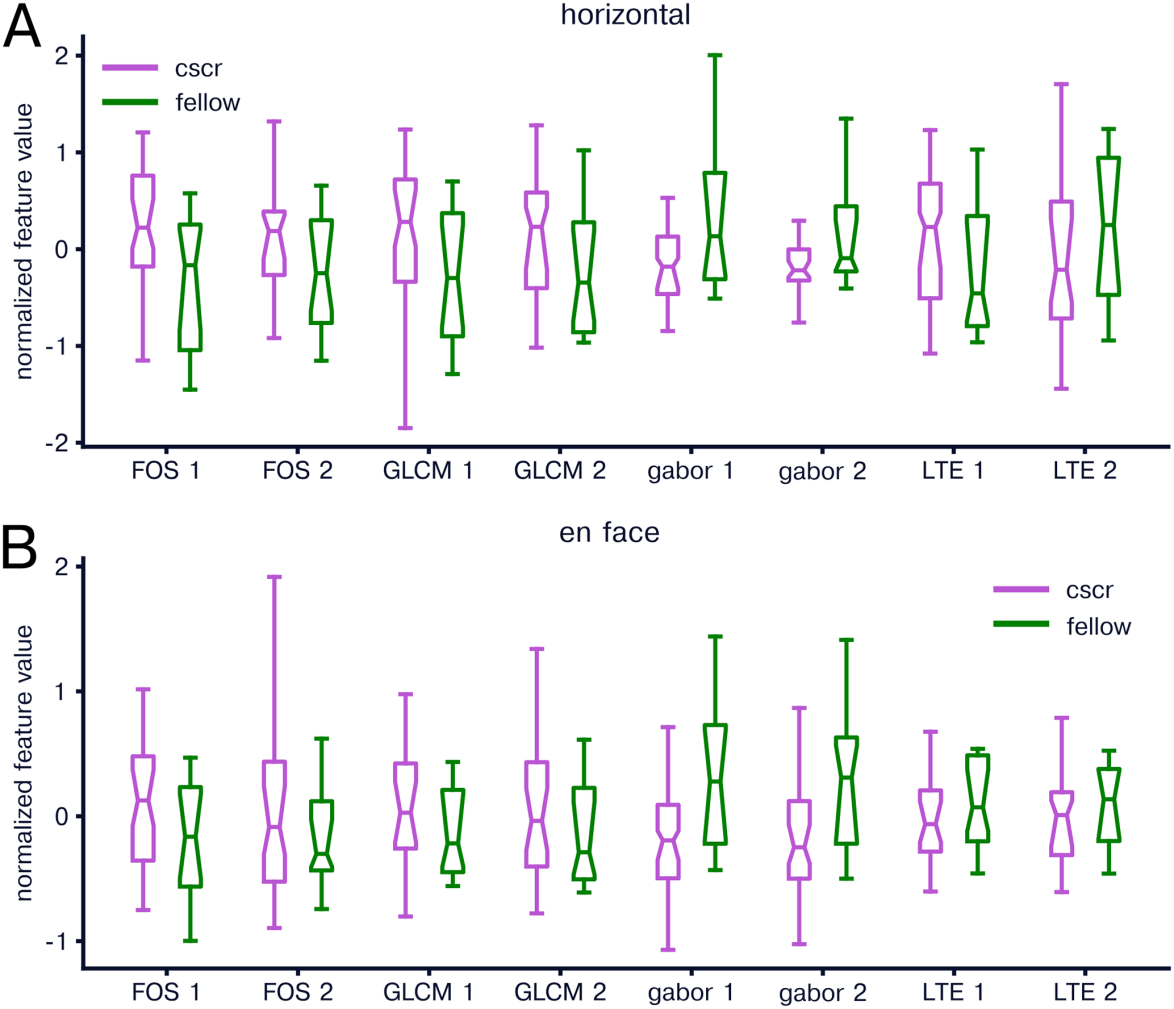
Comparison of radiomics features in CSCR and fellow eyes. (**A**) Same as in Figure 1A but comparing CSCR and fellow eyes. (**B**) Same as in Figure 1B but comparing CSCR and fellow eyes.

To evaluate the properties of fellow eyes, we first compared fellow eye features to healthy and CSCR eyes. We found significant differences between healthy and fellow horizontal images in eight of 16 FOS features, 10 of 14 GLCM features, 14 of 16 Gabor features, six of six LTE features (Fig. 5A). For en face images, we found significant results for 12 of 16 FOS features, 11 of 14 GLCM features, six of 16 Gabor features, five of six LTE features (Fig. 5B). We did not find any significant differences in individual features between CSCR and unaffected fellow eyes in any feature group for horizontal or en face images (Fig. 6). We next examined whether combined fellow eye features were more similar to healthy or CSCR features by training a logistic regression classifier to distinguish healthy and CSCR eyes and then input unaffected fellow eye images and measured the percentage of images that were classified as CSCR. For horizontal scan images 90.4% (90.2%–90.6%) were classified as CSCR and for en face images 90.2% (89.8%–90.2%) were classified as CSCR.

**Figure 7.**
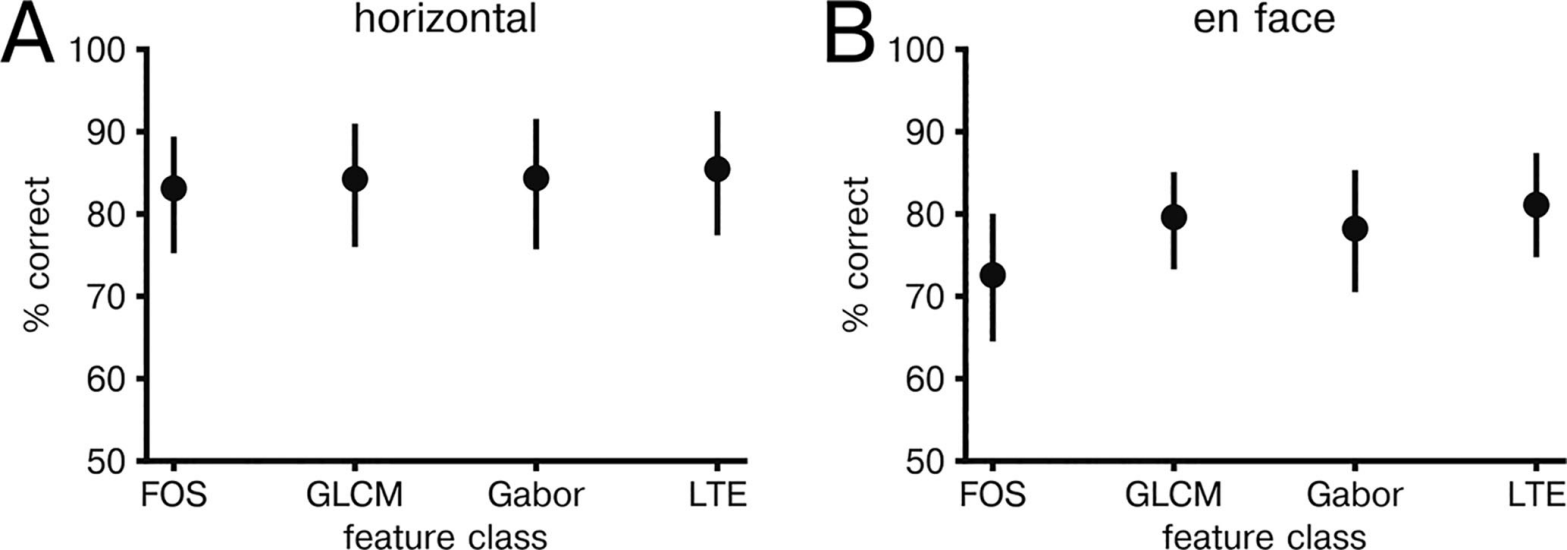
Classification of healthy vs fellow eyes by feature group. (**A**) Same as in Figure 2A but comparing healthy and fellow eyes. (**B**) Same as in Figure 2B but comparing healthy and fellow eyes.

**Figure 8.**
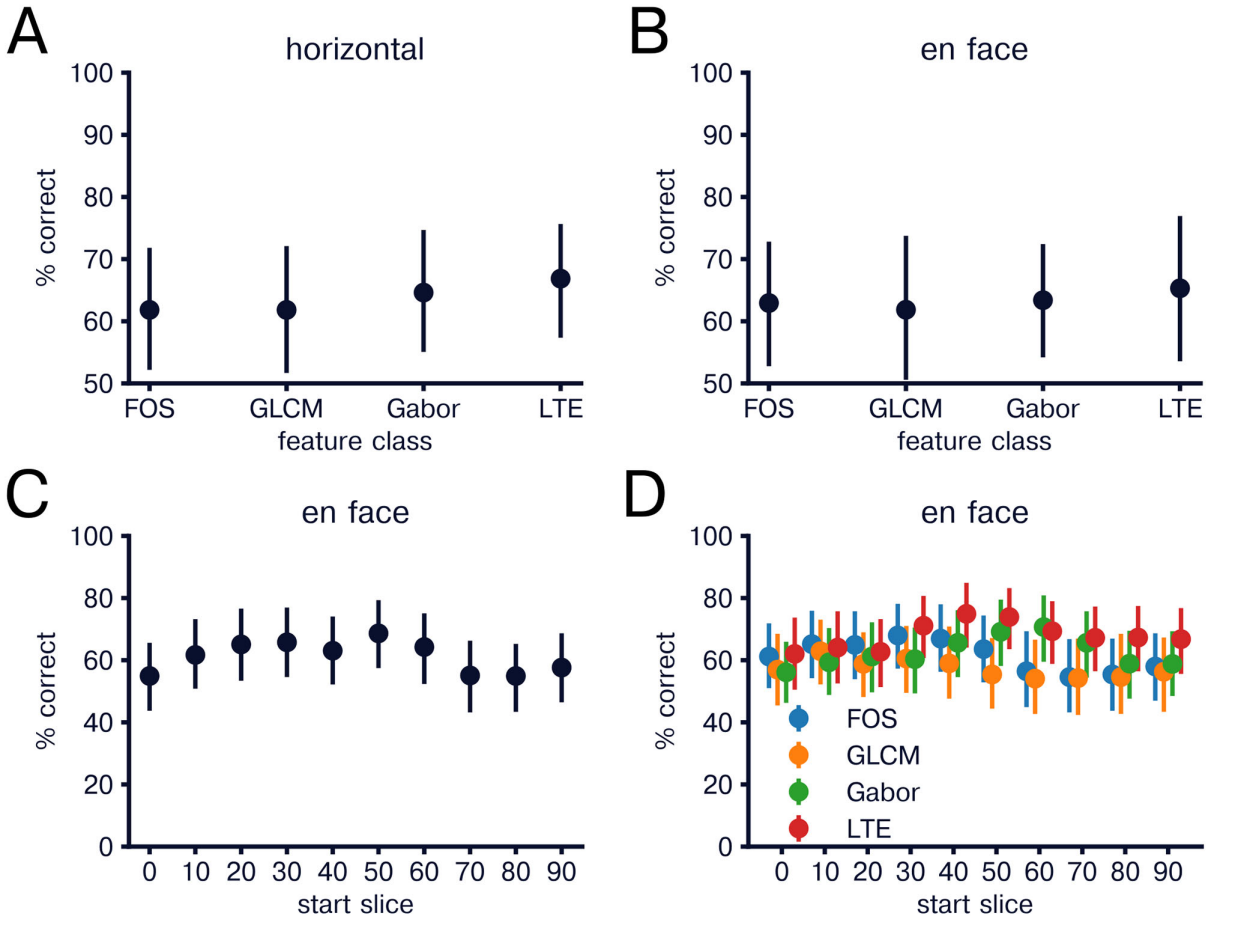
Classification of CSCR vs fellow by feature group and depth. (**A**) Same as in Figure 2A but comparing CSCR and fellow eyes. (**B**) Same as in Figure 2B but comparing CSCR and fellow eyes. (**C**) Same as in Figure 3A, but comparing CSCR and fellow eyes. (**D**) Same as in Figure 3B, but comparing CSCR and fellow eyes.

To further compare fellow eyes to healthy and CSCR eyes, we trained classifiers to distinguish healthy and fellow eyes and classifiers to distinguish CSCR and fellow eyes. Classifiers trained to distinguish healthy and fellow eyes achieved accuracy of 84.8% (76.0%–91.7%) and ROC-AUC of 0.922 (0.838–0.983) for horizontal images (Fig. 7A) and 87.5% (81.0%–92.9%) and ROC-AUC of 0.981 (0.948–1.00) with significant accuracy and ROC-AUC for all feature groups (*P* < 0.05; Fig. 7B). Classifiers trained to distinguish CSCR and fellow eyes resulted in accuracy of 62.9% (53.0–72.2%) and ROC-AUC of 0.651 (0.517–0.789) for horizontal images with only LTE subgroup classification reaching significant accuracy and ROC-AUC of 65.2% (50.3%–79.4%) and 0.669 (0.573–0.756), respectively (Fig. 8A). En face image accuracy was 60.7% (50.9%–70.0%) and ROC-AUC of 0.528 (0.371–0.678), with all image subgroups demonstrating similar results (Fig. 8B). However, when divided by 10% depth, en face images showed significant classification peaking at 50% depth with accuracy of 69.0% (58.6–78.5%) and ROC-AUC of 0.667 (0.541–0.819; Fig. 8C). Separating by feature groups showed significant peak for LTE features at 40% to 50% depth with accuracy of 74.9% (64.6%–84.1%) and ROC-AUC of 0.709 (0.587–0.833; Fig. 8D).

## Discussion

In this article, we extracted and compared radiomics features from OCT images of choroid obtained from healthy and CSCR eyes. We found significant differences in radiomics feature values between the two groups in both horizontal b-scan and en face images. Machine learning classification of radiomics features successfully differentiated healthy and CSCR eyes. Classification performance improved with en face depth within the choroid. We further extracted radiomics features from fellow eyes from CSCR patients and found that fellow eye features were more similar to CSCR eyes. However, we found that LTE features could be used to classify CSCR and fellow eyes with significant performance. Overall, this work demonstrates the effectiveness of radiomics feature analysis for evaluating choroidal pathology.

The identification of choroid biomarkers has the potential to improve the accuracy of clinical diagnosis, as well as to facilitate tracking of disease course over time. Previous work on choroidal biomarkers has predominantly focused on spatial biomarkers^5^^,^^6^ and on measures of choroid vascularity.^7^^,^^8^ These approaches have brought to light important features of choroid architecture that change in pathologic states. Despite these successes, the identification of additional biomarkers is labor intensive and relies on human insight, which may miss potentially fruitful markers. Our results demonstrate the potential of radiomics to accelerate this process by automating identification of clinically relevant biomarkers.

This article is, to our knowledge, the first to apply radiomics feature extraction to choroid OCT for the purposes of studying pathological states. Our literature review identified one other paper which utilized radiomics on OCT angiography to study the distribution of choroid radiomics features across healthy patients and concluded that features tended to be consistent across eyes.^10^ In the present study, we compared healthy eyes to eyes with CSCR and found significant differences between the two groups. Further work is needed to understand how these differences vary with clinical presentation and with treatment, as well as how radiomics features differ across other pathologic states.

Previous work has demonstrated that CSCR has the greatest effect on outer choroid layers compared to inner layers. For example, CSCR eyes display increased medium and large vessel size in Sattler's and Haller's layers, respectively.^1^ Middle and deep choroid also demonstrate increased choroidal vascularity index compared to inner choroid and compared to healthy individuals.^8^ In this study, we applied radiomics features to en face images in 10% depth increments, allowing us to compare features between healthy and CSCR eyes at varying choroid depths, supporting involvement of larger choroidal vessels in pachychoroid diseases. Overall, our results corroborate these previous findings and demonstrate the flexibility of a radiomics-based approach to identify subtle variation in choroidal architecture. Furthermore, our use of radiomics on en face images demonstrates the importance of examining local image features rather than summarizing entire images with a single value.

Unaffected fellow eyes in CSCR patients are known to demonstrate similar features to the pathologic eye at the level of the choroid. Previous work has demonstrated this relationship in terms of similarity of choroidal vascularity index and choroid thickness in CSCR and unaffected fellow eyes.^36^ When looking at horizontal b-scans our results largely agree with this previous work with radiomics signatures of CSCR identified in fellow eyes. However, in both horizontal and en face images we found that LTE features distinguished CSCR and fellow eyes. It is possible that these features represent biomarkers of conversion to a pathologic state and further work is needed to understand how these features change with disease course and with treatment.

Past studies have demonstrated the correlation of radiomics features with clinically relevant aspects of disease. For example, multiple studies have shown the utility of radiomics features for predicting response to anti-VEGF treatment in macular edema^24^ and in neovascular AMD^12^^,^^22^ and have demonstrated structural changes in pigment epithelial detachment in response to anti-VEGF treatment.^12^ Additional work has found correlations between radiomics features and pre-clinical diabetic retinopathy^23^ and cytokine levels in diabetic macular edema.^25^ Radiomics features have also been shown to differ between healthy eyes and eyes with severe myopic maculopathy.^26^ In the current study radiomics signatures of CSCR were identified in both pathologic and fellow eyes. Furthermore, our study demonstrates differences between CSCR and fellow eyes that may represent structural changes in the disease state. Further work is needed to better assess how radiomics features change over time with the natural course of disease and in response to treatment, as well as to understand how radiomics features correlate with a broader range of disease states.

Our study focused on radiomics as a feature extraction tool for biomarker identification. An alternative to this approach is feature extraction through learned transformations, as is done in most deep learning systems. One example of deep learning-based feature extraction is in the initial layers of a convolutional neural network in which patches of image pixels are transformed into feature values that are automatically optimized to maximize performance on a task. Such a custom-tailored approach can result in extremely high task performance, often exceeding that of human experts.^13^^,^^16^ However, this flexibility comes at a high cost in terms of the quantity of data and the number of computations required for training. In addition, because learned transforms vary by model or even between instantiations of the same model architecture, it is difficult to develop intuition for the meaning of the learned features. On the other hand, radiomics feature extraction requires relatively small amounts of data and fewer computations compared to deep learning models, and the static nature of the transforms allows for deeper study for building intuition.^12^ Importantly, however, deep learning–based feature extraction and radiomics feature extraction need not be viewed as mutually exclusive, and it is possible that future work may use hybrid models that leverage the advantages of both types of feature extraction approaches.

The main limitations of this study are the relatively small sample size and retrospective analysis. This study represents a proof of concept demonstration of radiomics as a tool for identifying pathology within choroid OCT. A larger study population will be needed before this technology can be applied in the clinical setting to ensure generalization of results. Despite this limitation, several steps were taken to improve generalizability. First, our radiomics analysis requires fewer parameters than other commonly used models because the radiomics features are standardized statistics rather than learned transformations. In addition, we used a simple linear classifier on the computed features, further limiting the number of model parameters. Together these design choices made our approach less prone to overfitting. Second, incorporating all B-scans into the training process rather than just foveal cross sections, maximized our use of the available data. At the same time, our leave-one-eye-out cross-validation approach ensured that training data was well-isolated from test data. Future work could further improve data utilization by focusing on local regions of the images that are more likely to demonstrate pathology.

Future work will focus on at least four areas that will expand the application of radiomics feature extraction. First, choroidal radiomics features need to be evaluated in a wider range of pathologic states. Second, radiomics features need to be correlated with clinical descriptions of image findings. Combined, these first two focus areas will both develop understanding of the clinical relevance of radiomics features while also identifying novel features that may be missed by human observers. Third, radiomics features need to be tracked over time and before and after treatment. Finally, radiomics features need to be validated using larger multicenter datasets in prospective studies before real-world clinical application.

Overall, this study has demonstrated the use of radiomics texture analysis to identify biomarkers that distinguish healthy and CSCR states in OCT. Layer-by-layer analysis of en face OCT images further demonstrated that these distinguishing features are most prominent in deep choroid in eyes with CSCR. Further study of radiomics texture features as potential biomarkers for measuring choroidal pathology has the potential to facilitate the development of novel clinical decision support tools for enhanced patient care across a spectrum of chorioretinal diseases.
